# Effect of aerosol particles generated by ultrasonic humidifiers on the lung in mouse

**DOI:** 10.1186/1743-8977-10-64

**Published:** 2013-12-21

**Authors:** Masakazu Umezawa, Keisuke Sekita, Ken-ichiro Suzuki, Miyoko Kubo-Irie, Rikio Niki, Tomomi Ihara, Masao Sugamata, Ken Takeda

**Affiliations:** 1 Department of Hygienic Chemistry, Faculty of Pharmaceutical Sciences, Tokyo University of Science, 2641 Yamazaki, Noda, Chiba 278-8510, Japan; 2 The Center for Environmental Health Science for the Next Generation, Research Institute for Science and Technology, Tokyo University of Science, 2641 Yamazaki, Noda, Chiba 278-8510, Japan; 3 Department of Pathology, Tochigi Institute of Clinical Pathology, 2308-3 Sasayama, Minami-akatsuka, Nogi-machi, Shimotsuga-gun, Tochigi 329-0112, Japan

## Abstract

**Background:**

Ultrasonic humidifiers silently generate water droplets as a cool fog and produce most of the dissolved minerals in the fog in the form of an aerosolized “white dust.” However, the health effect of these airborne particles is largely unknown. This study aimed to characterize the aerosol particles generated by ultrasonic humidifiers and to investigate their effect on the lung tissue of mice.

**Methods:**

An ultrasonic humidifier was operated with tap water, high-silica water, ultrapure water, or other water types. In a chamber (0.765 m^3^, ventilation ratio 11.5 m^3^/hr), male ICR mice (10-week-old) were exposed by inhalation to an aerosol-containing vapor generated by the humidifier. After exposure for 7 or 14 days, lung tissues and bronchoalveolar lavage fluid (BALF) were collected from each mouse and examined by microarray, quantitative reverse transcription-polymerase chain reaction, and light and electron microscopy.

**Results:**

Particles generated from the humidifier operated with tap water had a mass concentration of 0.46 ± 0.03 mg/m^3^, number concentration of (5.0 ± 1.1) × 10^4^/cm^3^, and peak size distribution of 183 nm. The particles were phagocytosed by alveolar macrophages in the lung of mice. Inhalation of particles caused dysregulation of genes related to mitosis, cell adhesion molecules, MHC molecules and endocytosis, but did not induce any signs of inflammation or tissue injury in the lung.

**Conclusion:**

These results indicate that aerosol particles released from ultrasonic humidifiers operated with tap water initiated a cellular response but did not cause severe acute inflammation in pulmonary tissue. Additionally, high mineral content tap water is not recommended and de-mineralized water should be recommended in order to exclude any adverse effects.

## Background

The indoor air environment is important for human health because humans spend most of their time indoors [1]. Volatile organic compounds [2], microorganisms [3], environmental allergens [4], and particle matter [5] have been reported as important factors of the indoor environment. It is necessary to conduct safety assessments of consumer products, e.g., air conditioners, humidifiers, and air purifiers, which are all prevalent in commercial facilities, hospitals, schools, and homes.

The most important factors of the indoor air environment are temperature and humidity [6,7]. Humidifiers are used to prevent excessive drying and to maintain comfortable room humidity. There are three types of humidifier: evaporative, steam, and ultrasonic vaporizers [8]. While ultrasonic humidifiers need little electricity to work and release cooler vapor than the steam-type, they must be cleaned to avoid bacterial contamination since the vapor contains all impurities that are present in the reservoir [9,10]. Only ultrasonic humidifiers release most of the dissolved and suspended components of the water, including microorganisms and pathogens, into the air [11]. Fatal pulmonary damage, i.e., rapidly progressive respiratory fibrosis, has also been reported when a water aerosol which contained biocides was released from humidifiers and was inhaled by humans [12].

Even if the water has no contamination by microorganisms or pathogens, ultrasonic humidifiers may exert some effects on human health. Previous studies showed that ultrasonic humidifiers may release dissolved minerals as an aerosol [13]. Highsmith et al. [14] reported that the fine particle concentration exceeded 6.3 mg/m^3^ when an ultrasonic humidifier was operated in a closed room. A subsequent study reported that the “white dust” induced lung injury [15]. However, the detailed components of the aerosol (or dust) and its health effect (e.g., inflammatory response in the lung) remained unknown. The aim of the present study was to characterize the aerosol particles generated by an ultrasonic humidifier and to investigate their effects on the lung in a mouse model.

## Methods

### Ultrasonic humidifier operation

An ultrasonic humidifier, BBH-07 (Hanwa Ltd., Osaka, Japan) was placed in each chamber with a volume of 0.765 m^3^ and a ventilation ratio of 11.5 m^3^/hr in the Center for Environmental Health Science for the Next Generation (Research Institute for Science and Technology, Tokyo University of Science, Noda, Chiba, Japan). The humidifier was operated at approximately 60 mL/hr liquid output rate with one of the following types of water: tap water obtained in Noda-city (Chiba, Japan), high-silica water purchased from Riken Mineral Kenkyusho K.K (Ebino, Miyazaki, Japan), a graded series of calcium chloride solution (4–400 mg/L of calcium), reverse osmotic membrane-filtration water (RO water) purchased from Ako Kasei Co., Ltd. (Ako, Hyogo, Japan), or an ultrapure water generated by an Automatic Sanitization Module (Merck Millipore, Billerica, MA, USA). Tap water and high-silica water were examples of drinking water containing minerals, while the calcium chloride solutions were used as a model of mineral-dissolved water with known concentrations. The concentration of Na, Ca, Mg, and Si in each type of water was measured using inductively coupled plasma mass spectrometry (ICP-MS) by Murata Keisokuki Service Co., Ltd. (Kanagawa, Japan). The humidifier was operated at approximately 80% power.

### Characterization of particles from the humidifier

The mass concentration of particles in the chamber was measured by a Piezobalance dust monitor Model 3521 (Kanomax Japan Inc., Osaka, Japan). The number concentration of particles was measured by a portable particle counter CPC 3007 (Tokyo Dylec Co., Tokyo, Japan), which can count particles with 10–1000 nm diameter. The size distribution of particles (10–410 nm) was measured by a switch-mode power supplies system Model 3936 (TSI Inc., Shoreview, MN, USA) composed of a classifier DMA3081 (TSI Inc.) and a condensation particle counter CPC 3785 (TSI Inc.) at a flow rate of 0.6 L/min.

Airborne particles in the chamber were classified using a cascade impactor (NL-3-2.5c0.5c, Tokyo Dylec). Fractions of 0.5 − 2.5 μm and <0.5 μm were collected for 30 sec and 15 min, respectively, at a flow rate of 0.3 L/min. The fractionated particles were collected on a silicon wafer (SI-500452, Nilaco Co., Tokyo, Japan) or a collodion film-patched mesh (200 mesh Cu; Nisshin EM Co., Tokyo, Japan) by electrostatic force using a sampler for suspended particle matter, SSPM-100 (Shimadzu Co., Kyoto, Japan). To determine the form and elemental component of the particles, they were observed by a field emission-type scanning electron microscope/energy-dispersive X-ray spectrometer (FE-SEM/EDS) (JSM-6500 F, JEOL Ltd., Akishima, Tokyo, Japan) with accelerating voltage of 15.0 kV and a transmission electron microscope (TEM: JEM-1200EX II, JEOL) with accelerating voltage of 90.0 kV.

### Exposure of mice to particles released from an ultrasonic humidifier

All animals were treated and handled in accordance with the national guidelines for care and use of laboratory animals and with the approval of the Tokyo University of Science Institutional Animal Care and Use Committee. Male ICR mice (10 weeks old) were purchased from Japan SLC Inc. (Hamamatsu, Shizuoka, Japan) and housed in a controlled room with *ad libitum* access to chow and water. After acclimation for 7 days, they were exposed by inhalation to an aerosol-containing vapor generated by an ultrasonic humidifier. The relative humidity was approximately 75% in the chamber with operating the humidifier. Tap water obtained in Noda-city, high-silica water, or ultrapure water were used to operate the ultrasonic humidifier for 7 days (8 hr/day [10:00 am − 6:00 pm] or whole day) or 14 days (whole day) (Table 1). After each exposure period, lung tissues and bronchoalveolar lavage fluid (BALF) were collected from each mouse under anesthesia by intraperitoneal injection of pentobarbital sodium.

**Table 1:** Experimental conditions for analysis of the effect of particles generated by ultrasonic humidifier on mouse lung

	**Exposure time**	**Tested water**
Experiment 1	8 hr/day, 7 days	Tap water/Ultrapure water
Experiment 2	8 hr/day, 7 days	High-silica water/Ultrapure water
Experiment 3	24 hr/day, 7 days	Tap water/Ultrapure water
Experiment 4	24 hr/day, 7 days	High-silica water/Ultrapure water
Experiment 5	24 hr/day, 14 days	High-silica water/Ultrapure water

### BALF cell analysis

The cells in BALF were collected by centrifugation at 1,000 × *g* for 10 min and resuspended in phosphate-buffered saline (pH 7.4). After counting the total number of cells, they were observed in a Giemsa-stained smear on a glass slide under a light microscope (BX51; Olympus Co., Tokyo, Japan). Statistical analysis was performed using an unpaired *t*-test and the level of significance was set at P < 0.05.

### Total RNA isolation

Lung tissue was homogenized in Isogen (Nippon Gene Co., Ltd., Tokyo, Japan). Total RNA was isolated by chloroform, purified by isopropanol, precipitated in 70% ethanol according to the manufacturer’s protocol and then suspended in RNase-free water. The RNA quantity was determined by absorption spectrophotometry at OD260 in a BioPhotometer plus (Eppendorf, Hamburg, Germany). Isolated RNA from each sample was provided for quantitative RT-PCR and microarray analyses.

### Complementary DNA microarray

Total RNAs (*n* = 4 − 5/group) were pooled (45 μg) for each group and purified by RNeasy Micro Kit (Qiagen, Hilden, Germany). The integrity of RNA was evaluated by Bioanalyzer 2100 (Agilent Technologies Inc., Santa Clara, CA, USA). Complementary-DNA (cDNA) of each of the RNA samples was labeled by Cy3 and hybridized to the SurePrint G3 Mouse Gene Expression 8 × 60 K Microarray (Agilent Technologies) consisting of 62976 spots (containing probes for 24163 mRNAs) according to the protocol of Oncomics Co. Ltd. (Nagoya, Aichi, Japan). The microarray was then washed using Gene Expression Wash Pack (Agilent Technologies) and scanned by a DNA Microarray Scanner (Agilent Technologies). Scanner output images were normalized and digitalized by Feature Extraction software (Agilent Technologies) according to the Minimum Information About a Microarray Experiment (MIAME) guidelines [16].

### Hierarchical cluster analysis

To extract gene sets for which differential expression was induced by airborne particles generated by the ultrasonic humidifier, microarray data from experiments 1–5 (Table 1) were hierarchically clustered using a complete linkage algorithm and Euclidean distance as the distance metric [17]. The analysis was performed using Cluster 3.0 [18] and the result was visualized by Java TreeView [19].

### Functional analysis of microarray data

To better understand the biological meaning of the microarray results, functional analysis was performed using gene annotation by gene ontology (GO) and pathway. Genes were annotated with GO and pathway using an annotation file (gene2go.gz) provided by the National Center for Biotechnology Information (NCBI; Bethesda, MD, USA) [20] and c2.cp.v3.1.symbols.gmt by the Broad Institute (Cambridge, MA, USA) [21]. The annotations were updated on March 6, 2013. Enrichment factors for each GO and pathway were defined as (*nf*/*n*)/(*Nf/N*), where *nf* is the number of flagged (differentially expressed) genes within the category, *Nf* is the total number of genes within that same category, *n* is the number of flagged genes on the entire microarray, and *N* is the total number of genes on the microarray. Statistical analysis was done with Fisher’s exact test based on a hypergeometric distribution and then the GO and pathways with enrichment factors ≥3, *nf* ≥3 and *P* <0.01 were extracted.

### Quantitative RT-PCR

Total RNA (1 μg) for each sample was treated by Dnase (Promega Co., Fitchburg, WI, USA) and then by M-MLV reverse transcriptase (Invitrogen Co., Carlsbad, CA, USA) to obtain first strand cDNA according to the manufacturer’s instructions. Quantitative PCR was performed in a 96-well plate and an Mx3000P (Agilent Technologies) with SYBR Green Realtime PCR Master Mix (Thunderbird; Toyobo Co., Ltd., Osaka, Japan) and specific primers (Fasmac Co. Ltd., Atsugi, Kanagawa, Japan) or with Probe qPCR Master Mix (Thunderbird; Toyobo Co.) and primer (Fasmac)/probe (Biosearch Technologies Japan, Inc., Tokyo, Japan) sets for indicated genes (Additional file 1: Table S1). Statistical analysis was performed using an unpaired *t*-test and the level of significance was set at P < 0.05.

### Immunohistochemistry

Lung tissue was fixed in phosphate-buffered (pH7.4) 4% formaldehyde, and then cryoprotected in phosphate-buffered 30% sucrose solution for 24–48 hr. Next, brains were frozen and cut into 4-μm sections using a cryostat (Tissue-Tek Cryo; Sakura Finetek Japan). Immunohistochemical visualization of a macrophage marker, F4/80, was performed using antibodies and avidin − biotin-peroxidase methods. After blocking endogenous peroxidase and preincubation with 10% normal horse serum, sections were treated overnight with primary rat monoclonal anti-F4/80 antibody (ab6640; Abcam plc, Cambridge, UK), secondary biotinylated donkey anti-rat IgG (AP189B; Merck Millipore) for 2 hr, and finally treated with an avidin − biotin-peroxidase complex (Vectastain ABC peroxidase kit, Vector Laboratories, CA, USA) for 4 hr. Sections were then reacted for peroxidase activity in a solution of 0.04% 3,3′-diaminobenzidene (DAB) in 0.1 M Tris–HCl buffer (pH 7.6) and 0.01% hydrogen peroxide water. Immunoreactive cells on sections were observed under BX51 light microscope. Quantitative analysis was performed on all sections. Statistical analysis was performed using an unpaired *t*-test and the level of significance was set at P < 0.05.

### Electron microscopic analysis

Lung tissues and BALF cells were prefixed in cacodylate-buffered 2.5% glutaraldehyde (pH 7.4) for 24 hr, washed in cacodylate buffer, and postfixed with 2% osmium tetroxide (Nisshin EM) for 1 hr. After washing in cacodylate buffer, the tissue samples were dehydrated using a graded series of ethanol (up to 100%) and propylene oxide (Nisshin EM), and then embedded in Quetol 812 (Nisshin EM). Ultra-thin sections (80-nm thick) were cut on the ultra-microtome EMUC6 (Leica Microsystems K.K., Tokyo, Japan). Some sections were double-stained with uranyl acetate and lead citrate. They were then observed under the TEM (JEM-1200EX II) with accelerating voltage of 80–90 kV.

## Results

### Mineral and silica concentration of each water type

Tap water, high-silica water, RO water, and ultrapure water were subjected to ICP-MS analysis to measure the concentrations of Na, Ca, Mg, and Si (SiO_2_: hydrated silica) (Table 2). Tap water contained 15–25 mg/L Na, 15–30 Ca, 4–7 Mg and 20–50 SiO_2_. High-silica water contained 73 mg/L SiO_2_. Most of the minerals and silica were absent in RO water, which contained only (3 mg/L) Na. No minerals and silica were detected in ultrapure water.

**Table 2:** Mass concentration of mineral and silica in each type of water

	**Na (mg/L)**	**Ca (mg/L)**	**Mg (mg/L)**	**SiO**_**2 **_**(mg/L)**	**Total (mg/L)**
Tap water	18	25	5.6	24	73
High-silica water	15	12	3.7	73	104
RO water	3	<0.1	<0.1	<0.1	3
Ultrapure water	<0.1	<0.1	<0.1	<0.1	<0.1

### Concentration and size distribution of particles released from humidifier

**Figure 1:**
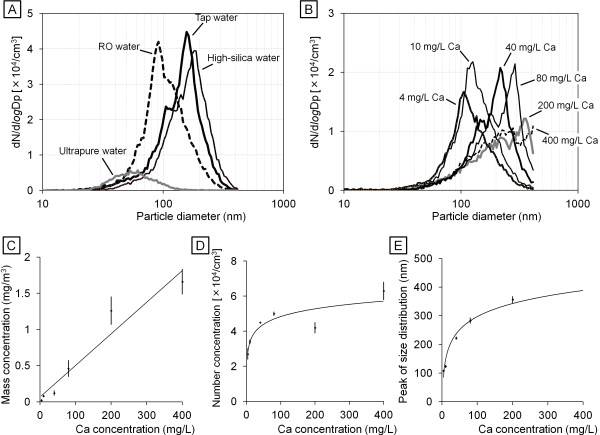
**Size distribution of airborne particle released from ultrasonic humidifier with each water type.** Number distribution of the size of particles from humidifier with **(A)** tap water, high-silica water, ultrapure, and **(B)** serial concentrations of calcium chloride solution are shown. The correlation of calcium chloride concentration with **(C)** mass concentration, **(D)** number concentration, and **(E)** peak size distribution are also shown.

First, we determined the mass and number concentrations and the peak size distribution of particles generated by an ultrasonic humidifier that was operated with each type of water in our chamber (Table 3). The results with tap water (Noda-city, and other area for which data not shown) were: mass concentration 0.35 − 0.50 mg/m^3^, number concentration 3.5 − 7.5 × 10^4^ particles/cm^3^, and 155–195 nm peak size distribution (number distribution). The results obtained with high-silica water were similar to tap water. The mass concentration of particles generated by the humidifier with RO water was below the detection limit (<0.01 mg/m^3^) but the number concentration was similar to tap water. The size distribution pattern with tap water and high-silica water was similar to a previous report [22] (Figure 1A). The humidifier generated visible fog (micro-sized water droplets) and submicro-sized residuals; however, because the SMA-CPC system could detect particles with diameter 10–410 nm, we characterized only the residuals as particles but not obtain size distribution data of the primary water droplets of the humidifier. The size of the particles generated by the humidifier using RO water was smaller to that of water types which contain a higher concentration of minerals and silica, e.g., tap water and high-silica water. The fog from the humidifier with ultrapure water did not contain non-volatile particles nor did it elicit any peak size distribution. The result with another type of pure water which originated from a PURELAB Option-7/15 Water Purification System (ELGA LabWater, Bucks, UK) was very similar to that with the ultrapure water (data not shown).

**Table 3:** Concentration and size of airborne particles released from ultrasonic humidifier for each type of water

**Used water**	**Mass concentration (mg/m**^**3**^**)**	**Number concentration (#/cm**^**3**^**)**	**Peak of size distribution (nm)**
Tap water	0.46 ± 0.03	(5.0 ± 1.1) × 10^4^	183 ± 9
High-silica water	0.58 ± 0.04	(3.6 ± 0.0) × 10^4^	180 ± 3
CaCl_2_ (400 mg/L Ca)	1.66 ± 0.16	(6.3 ± 0.5) × 10^4^	>410
CaCl_2_ (200 mg/L Ca)	1.26 ± 0.19	(4.2 ± 0.3) × 10^4^	356 ± 11
CaCl_2_ (80 mg/L Ca)	0.46 ± 0.11	(5.0 ± 0.1) × 10^4^	283 ± 9
CaCl_2_ (40 mg/L Ca)	0.12 ± 0.03	(4.5 ± 0.0) × 10^4^	222 ± 4
CaCl_2_ (10 mg/L Ca)	0.08 ± 0.01	(3.4 ± 0.1) × 10^4^	123 ± 4
CaCl_2_ (4 mg/L Ca)	0.02 ± 0.01	(2.7 ± 0.3) × 10^4^	108 ± 2
RO water	<0.01	(4.4 ± 0.6) × 10^4^	90 ± 2
Ultrapure water	<0.01	(0.7 ± 0.0) × 10^4^	Not determined

Data are shown as mean ± SD, calculated from 5 repeats of 2 min analyses.

To investigate the correlations between mineral concentration in water and each parameter of generated particle, we characterized the particles released from the ultrasonic humidifier using water containing serial concentrations of calcium chloride solution (4–400 mg/L Ca) (Table 3). The concentration of calcium chloride in water was positively correlated with the mass and number concentrations (Figure 1C and D) and the peak size distribution (Figure 1B and E) of generated particles. The correlation of the concentration of dissolved material in water with the mass concentration of particles generated was linear (r =0.97) (Figure 1C), whereas the correlation with the number concentration was logarithmic (r =0.98) (Figure 1D). These correlations were also observed in particles from the humidifier with serial concentrations of sodium chloride solution (4–400 mg/L Na) (data not shown). The mass concentration of the aerosol particles was also correlated with total mineral concentration of tap water, high-silica water and RO water (Figure 1A).

### Form and elemental composition of particles released from humidifier

**Figure 2:**
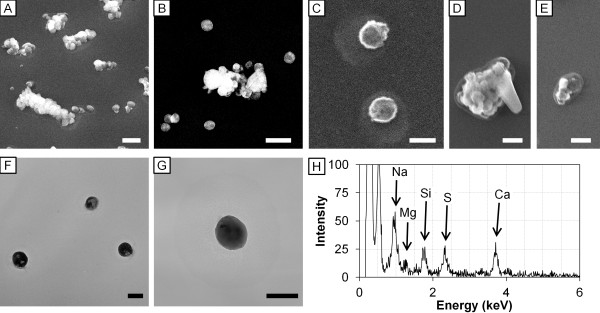
**FE-SEM images, TEM images and EDS spectrum of particles released from ultrasonic humidifier.** FE-SEM images of particles (0.5 − 2.5 μm) released from the humidifier with **(A)** tap water and **(B)** high-silica water under humidity 75%; particles (<0.5 μm) from the humidifier with tap water under **(C)** humidity 75% and **(D, E)** 99% are shown. TEM image of particles (<0.5 μm) from the humidifier with **(F)** tap water and **(G)** high-silica water under humidity 75%. Scale bars represent **(A, B)** 1 μm, **(C, F, G)** 200 nm, and **(D, E)** 500 nm. **(H)** EDS spectrum of the particle **(C)** is shown. The peaks of Na, Mg, Si, S, and Ca were detected at 0.97, 1.26, 1.78, 2.33, and 3.71 keV, respectively.

FE-SEM images showed that the humidifier operated with tap water or high-silica water released a large number of spherical-formed amorphous <0.5 μm particles and 0.5 − 2.5 μm agglomerates (Figure 2A − C). The particles were deformed under extremely high humidification (Figure 2D, E). TEM images also showed that the form of particles from the humidifier operated with tap water (Figure 2F) or high-silica water (Figure 2G) were spherical. The electron density of the particles was inhomogeneous under TEM observation, indicating that the particles were composed of multiple elements. EDS analysis showed the particles derived from tap water were composed of Na, Mg, Si, S (sulfate), and Ca, which were contained in tap water (Figure 2H).

### Effect of particles released from ultrasonic humidifier on mRNA expression in the lung

**Figure 3:**
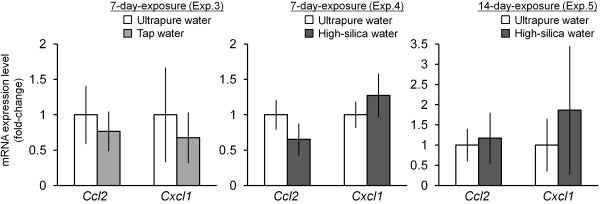
**Effect of inhalation of particles released from ultrasonic humidifier on mRNA expression of *Ccl2*, and *Cxcl1 *in the lung.** Expression levels of *Ccl2* and *Cxcl1* in the lung tissues (Experiments 3–5, Table 1) were analyzed by an Mx3000P (Agilent Technologies) with SYBR Green Realtime PCR Master Mix (Toyobo). Relative expression levels of the target genes were calculated for each sample after normalization against *Gapdh*. Statistical analysis was performed using an unpaired *t*-test and the level of significance was set at P < 0.05.

Next, we conducted microarray analysis to investigate the effect of particles from the humidifier on the lung. The effect of the particles was determined by mRNA expression of tested water groups compared to that of ultrapure water groups in each experiment (Table 1). A total of 15984 mRNAs were detected with quantitative fluorescence signals from the lung tissue samples. Hierarchical clustering revealed that 429 mRNAs were differentially expressed (238 upregulated and 191 downregulated) by particles derived from tested water (tap water or high-silica water) (Additional file 1: Figure S1). Expression change of some genes was correlated with the dose in the experiment 2, 4 and 5 using high-silica water. These 429 genes were enriched in 17 GO (Table 4) and 12 pathways (Table 5). The largest GO and pathway were mitosis (17 genes) and cell adhesion molecules (8 genes) (Additional file 1 Table S2). Among the 8 genes in cell adhesion molecules, upregulated *H2-DMb1* and downregulated *H2-Ab1*, *H2-Eb1*, *H2-Q2* were associated with major histocompatibility complex (MHC) molecules, and endocytosis followed by antigen processing. Quantitative RT-PCR data showed that expression levels of a monocyte chemoattractant chemokine *Ccl2* and a chemokine related to inhalation of silica particles *Cxcl1*[23] were not affected by the particles released from the ultrasonic humidifier operated with tap water or high-silica water (Figure 3). Expression of an inflammatory cytokine *Tnf* was at quite low level in the lung of any groups.

**Table 4:** Significantly enriched GO categories from the microarray data

**Gene ontology**	**Enrichment factor**	**P-value**
Mitosis	3.05	<0.001
Meiosis	5.35	0.001
Regulation of axonogenesis	8.61	0.001
Antigen processing and presentation of peptide or polysaccharide antigen via MHC class II	13.7	0.001
Chromosome, centromeric region	3.50	0.001
Kinetochore	3.80	0.001
Decidualization	12.2	0.002
MHC class II protein complex	12.2	0.002
Peptide antigen binding	12.2	0.002
Platelet activation	7.32	0.002
Gamma-aminobutyric acid signaling pathway	11.0	0.002
Motile cilium	6.65	0.003
Retinol metabolic process	9.98	0.003
Adenylate cyclase-activating G-protein coupled receptor signaling pathway	6.36	0.003
Antigen processing and presentation of exogenous peptide antigen via MHC class II	8.44	0.005
Chromosome segregation	3.60	0.006
Axon part	7.32	0.007

**Table 5:** Significantly enriched pathways from the microarray data

**Pathway**	**Enrichment factor**	**P-value**
CENP-A NAC-CAD complex (MIPS)	16.2	<0.001
PLK1 pathway (PID)	6.85	<0.001
Asthma (KEGG)	12.1	0.002
Cell adhesion molecules (KEGG)	3.53	0.002
Viral myocarditis (KEGG)	4.31	0.003
Allograft rejection (KEGG)	5.94	0.004
Mitotic prometaphase (Reactome)	3.66	0.006
Autoimmune thyroid disease (KEGG)	5.40	0.006
Graft versus host disease (KEGG)	5.40	0.006
GA13_PATHWAY (STKE)	5.24	0.007
Antigen processing and presentation (KEGG)	3.98	0.008
Type I Diabetes Mellitus (KEGG)	4.95	0.008

Contributors for curation: KEGG, KEGG: Kyoto Encyclopedia of Genes and Genomes; MIPS, MIPS database from CORUM (the Comprehensive Resource of Mammalian protein complexes); PID, Pathway Interaction Database (National Cancer Institute and Nature Publishing Group); STKE, Signal Transduction Knowledge Environment.

### Effect of particles released from ultrasonic humidifier on histology of the lung and alveolar macrophage

**Figure 4:**
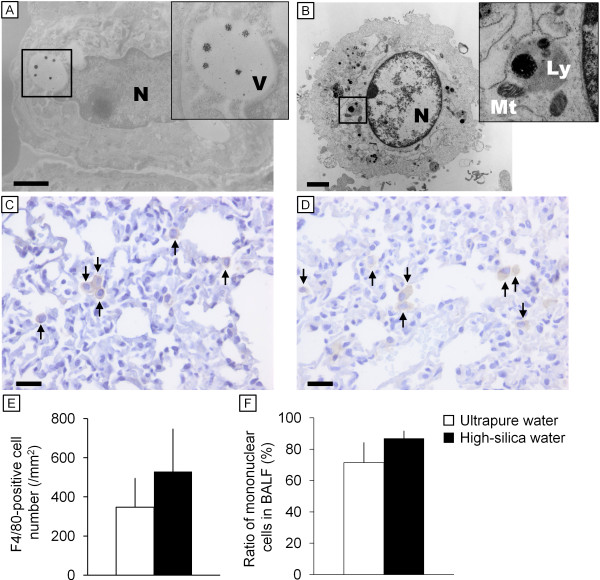
**Images of electron microscopy and immunohistochemistry of cells in the lung tissue and bronchoalveolar lavage fluid.** Transmission electron microscope images of macrophages in **(A)** lung tissue and **(B)** bronchoalveolar lavage fluid (BALF) of mouse of the 14-day high-silica water group (Experiment 5) are shown. The cells contained particle-like substances. Immunohistochemical F4/80 images of the lung tissue of **(C)** the ultrapure water group (control) and **(D)** the high-silica water group (14-day exposure, Experiment 5) are also shown. Arrows indicate F4/80 positive cells (macrophages). Mean number of F4/80-positive cells (±SD) is shown in **(E)**. There was no significant difference in the number of F4/80-positive cells in the lung between the ultrapure water and the high-silica water groups. **(F)** Ratio of mononuclear cells in BALF from Experiment 5 (14-day inhalation; *n* = 4/group). Scale bars represent **(A, B)** 1 μm and **(C, D)** 20 μm. Abbreviation: Ly, lysosome; Mt, mitochondria; N, nucleus; V, cytoplasmic vesicle.

HE-stained images did not show any remarkable changes or cell proliferation in lung after inhalation of particles from the humidifier with tap water or high-silica water (24 hr/day, 7–14 days). The lung tissues obtained from experiment 5 (24 hr/day for 14 days inhalation with the commercial high-silica water *vs.* ultrapure water; Table 1) were also subjected to analysis by immunohistochemistry and TEM. TEM observation of the lung tissues (Figure 4A) and BALF cells (Figure 4B) revealed that alveolar macrophages endocytosed the particles. The endocytosed particles showed a high contrast without staining by uranyl acetate and lead citrate (Figure 4A, insert), indicating that the particles were mineral particles rather than endogenous biomolecules. The diameter of the particles deposited in the lung was mostly within the range of 100–200 nm (Figure 4B, insert), and dissolving particles with approximately 20–60 nm diameter were also found in intracellular vesicles in the macrophage (Figure 4A, insert). The number of F4/80 positive cells (macrophages) did not significantly differ between ultrapure water and high-silica water groups by 14-day inhalation of the particles (Figure 4C − E). Abnormal macrophage accumulation was not observed in the lung of treated group. Although the total number of BALF cells was not affected by inhalation of particles from the humidifier with high-silica water (data not shown), the ratio of mononuclear cells in BALF tended to be increased (*P* =0.08) by 14-day inhalation of the particles (Figure 4F).

## Discussion

Tap water contains dissolved solid composed of calcium, sodium, other minerals and anions. The secondary water quality standard of total dissolved solids (TDS) is established as 500 mg/L by the Ministry of Health, Labour and Welfare (Japan) and the Environmental Protection Agency (US). Water also has ‘hardness’, which is definable as the concentration of resistant solid matter determined as the equivalent concentration of calcium carbonate. Since the concentration and size distribution of particles from ultrasonic humidifier were well correlated with the concentration of minerals, they can be estimated by the TDS and hardness of water provided to the humidifier.

Humidifiers are usually operated with tap water, and in these experiments we found that operation with tap water generated submicron-sized particles (100–1000 μm), which contribute to the mass concentration, and that the humidifier released them into the air. In contrast, the particles from the humidifier operated with water containing a low concentration of minerals (4 mg/L Na or Ca) were mostly nano-sized (<100 nm); therefore they had only a small mass (<0.03 mg/m^3^) but a large number concentration (>2 × 10^4^/cm^3^). This is the first study showing the characteristics of particles from the humidifier with tap water as well as other water with a low concentration of minerals. In the study using a series of calcium chloride solutions, the mass and the number concentrations were not well correlated. We concluded the cause of this worse correlation was another correlation between the mineral concentration of water and the particle size; i.e., the size was larger when the humidifier was operated with a water with higher concentration of minerals. Our data for the concentration of particles from ultrasonic humidifiers were measured in an experimental chamber (0.765 m^3^, ventilation ratio 11.5 m^3^/hr), whereas 0.59 mg/m^3^, which is much greater than daily PM_2.5_ standard (35 μg/m^3^), was also reported when the humidifier was operated in an actual residence environment with tap water containing 303 mg/L TDS [14].

The health effect of airborne particles is one of the main issues in environmental toxicology. The size distribution data on the fog also indicated that it substantially contained submicron-sized particles, which can pass the nasal airways and penetrate down to the lung [24]. Since a case report describing lung injury by “white dust” [15] suggested that the particles from ultrasonic humidifiers may exert adverse effects on the lung, we examined the effects in detail using a mouse model. Although the bacterial contamination was not investigated in the present study, chlorine-disinfected tap water, a commercial mineral water for drinking, and purified water were used for the experiments to minimize the contamination. In general, the insoluble component of the particles are not dissolved and deposit in pulmonary surfactant, where they are taken up by alveolar macrophages [25,26]. The inhaled particles taken up by the macrophages are transferred to intracellular vesicle and phagolysosome [27], exposed to acidic milieu, and can cause lysosome destabilization leading to inflammation [28]. TEM observation in the present study showed that the particles were being dissolved in the phagosome, and also indicated that the particles generated by the ultrasonic humidifier disintegrated in the phagolysosome of the macrophages. This observation was consistent with our microarray data suggesting that genes dysregulated by the particles from the ultrasonic humidifier were enriched in GO categories associated with MHC molecules and endocytosis. However, expression of *H2-Eb1* was suppressed by inhalation of particles from the humidifier with tap water or high-silica water. The result was in opposition to the effect of pulmonary exposure (intratracheal instillation) to titanium dioxide nanoparticles [29] and fullerenes [30] on the lung, suggesting that 7- and 14-day inhalation of the particles from the humidifier induced a cellular response from the alveolar macrophages but did not cause acute or sub-acute toxic effects on the lung tissue. This observation was supported by RT-PCR data suggesting that no significant difference in *Ccl2*, *Cxcl1*, and *Tnf* expression between the tap water or high-silica water group and each control (ultrapure water) group. A previous study showed that inhalation of silica-coated nanoparticles (10 mg/m^3^; 2 hr/day for 4 days) enhanced *Cxcl1* and *Tnf* expression in mouse lung [23]. Even though the total exposure period in the present study was longer than this previous study, the concentration of airborne particles containing silica from the humidifier was lower. The genes differentially expressed by inhalation of particles from the humidifier were enriched in mitosis, meiosis and related GO and pathways. However, histopathology (including cell proliferation) and macrophage location were not affected by the particles derived from tap water or high-silica water. Our data on the mononuclear cells in BALF indicated that alveolar macrophages tended to be activated by the aerosol particles. The results suggested that 7- and 14-day inhalation of the particles from the humidifier did not cause acute or sub-acute toxic effects on the lung of mice without pre-existing respiratory infection, disease or malfunction.

## Conclusion

The present study showed the characteristics of particles generated from ultrasonic humidifiers operated with tap water, a commercial mineral (high-silica) water, and other types of water. This study also showed the effect of 7- and 14-day inhalation of particles released from the humidifier on the lung in a mouse model. The particles were composed of multiple elements including sodium, magnesium, silicate, sulfate, and calcium. Mass and number concentrations and the peak size of the particles were positively correlated to concentration of dissolved mineral components in water provided to the humidifier. Inhalation of particles caused dysregulation of genes related to mitosis, cell adhesion molecules, MHC molecules and endocytosis followed by antigen processing, but did not induce any signs of inflammation or tissue injury in the lung. We conclude that the particles released from the humidifier operated with tap water and commercial mineral water induced a cellular response from the alveolar macrophages but did not cause acute or sub-acute toxic effects on the pulmonary organs in the mouse model. Finally, since the mass concentration of particles generated is linearly correlated with the concentration of dissolved material in water, cautions may be needed to use the humidifier in some areas with hard water. Long exposure to the aerosol particle from the humidifier may occur in home and working environment. High mineral content tap water is not recommended and de-mineralized water should be recommended in order to exclude any adverse effects.

## Abbreviations

BALF: Bronchoalveolar lavage fluid; cDNA: Complementary DNA; EDS: Energy-dispersive X-ray spectrometer; FE-SEM: Field emission-type scanning electron microscope; GO: Gene ontology; ICP-MS: Inductively coupled plasma mass spectrometry; RO: Reverse osmotic; TDS: Total dissolved solids; TEM: Transmission electron microscope.

## Competing interests

The authors declare that they have no competing interests.

## Authors’ contributions

KT is the main project leader. KT and MU conceived the overall research idea. KSe mainly performed all experiment procedures and data analyses. MU, KSu, MK-I, TI and MS were substantially involved in conducting the experiments and data analyses. MU mainly conducted microarray data analysis. KSu, a specialist in combining nanotechnology and biology, analyzed airborne particles by electron microscopy with KSe. MK-I, an expert on electron microscopy, analyzed particles in the tissue sections with KSe and MU. TI and MS conducted data analyses with pathological and clinical viewpoints. MU and KSe drafted the manuscript. All authors read and approved the final manuscript.

## Supplementary Material

Additional file 1: Table S1 Primer and probe sequences for quantitative RT-PCR. **Figure S1.** Profile of gene expression in the lung exposed to aerosol released from ultrasonic humidifier with tap water, high-silica water, or ultrapure water. **Table S2.** Differentially expressed genes with relevant enriched GO and pathways. Click here for file
