# Supplementary material for: Effect of aerosol particles generated by ultrasonic humidifiers on the lung in mouse
Source: Part Fibre Toxicol. 2013 Dec 21;10:64. doi: 10.1186/1743-8977-10-64 (PMC3922954; doi:10.1186/1743-8977-10-64)
Supplement: Additional file 1: Table S1 — Primer and probe sequences for quantitative RT-PCR. Figure S1. Profile of gene expression in the lung exposed to aerosol released from ultrasonic humidifier with tap water, high-silica water, or ultrapure water. Table S2. Differentially expressed genes with relevant enriched GO and pathways. [file 1743-8977-10-64-S1.pdf]

Supplementary Table S1 − Primer and probe sequences for quantitative RT-PCR

Gene	Sequence (5’ -> 3’)	Tm (°C)
Ccl2 (NM_011333.3)	F: GCTGGAGCATCCACGTGTT<br>R: TGGTGAATGAGTAGCAGCAGGT<br>P: CTCAGCCAGATGCAGTTAACGCCCC	60
Cxcl1 (NM_008176.3)	F: ACCGAAGTCATAGCCACACTC<br>R: CTCCGTTACTTGGGGACACC	60
Gapdh (NM_008084.2)	F: *TGTGCAGTGCCAGCCTCGTC*<br>R: GGATGCATTGCTGACAATCT	60
Tnf (NM_013693.2)	F: GAACTTCGGGGTGATCGGT<br>R: GTGAGGGTCTGGGCCATAGA<br>P: CAAAGGGATGAGAAGTTCCCAAATGGCC	60

Supplementary Figure S1 − Profile of gene expression in the lung exposed to aerosol released from ultrasonic humidifier with tap water, high-silica water, or ultrapure water

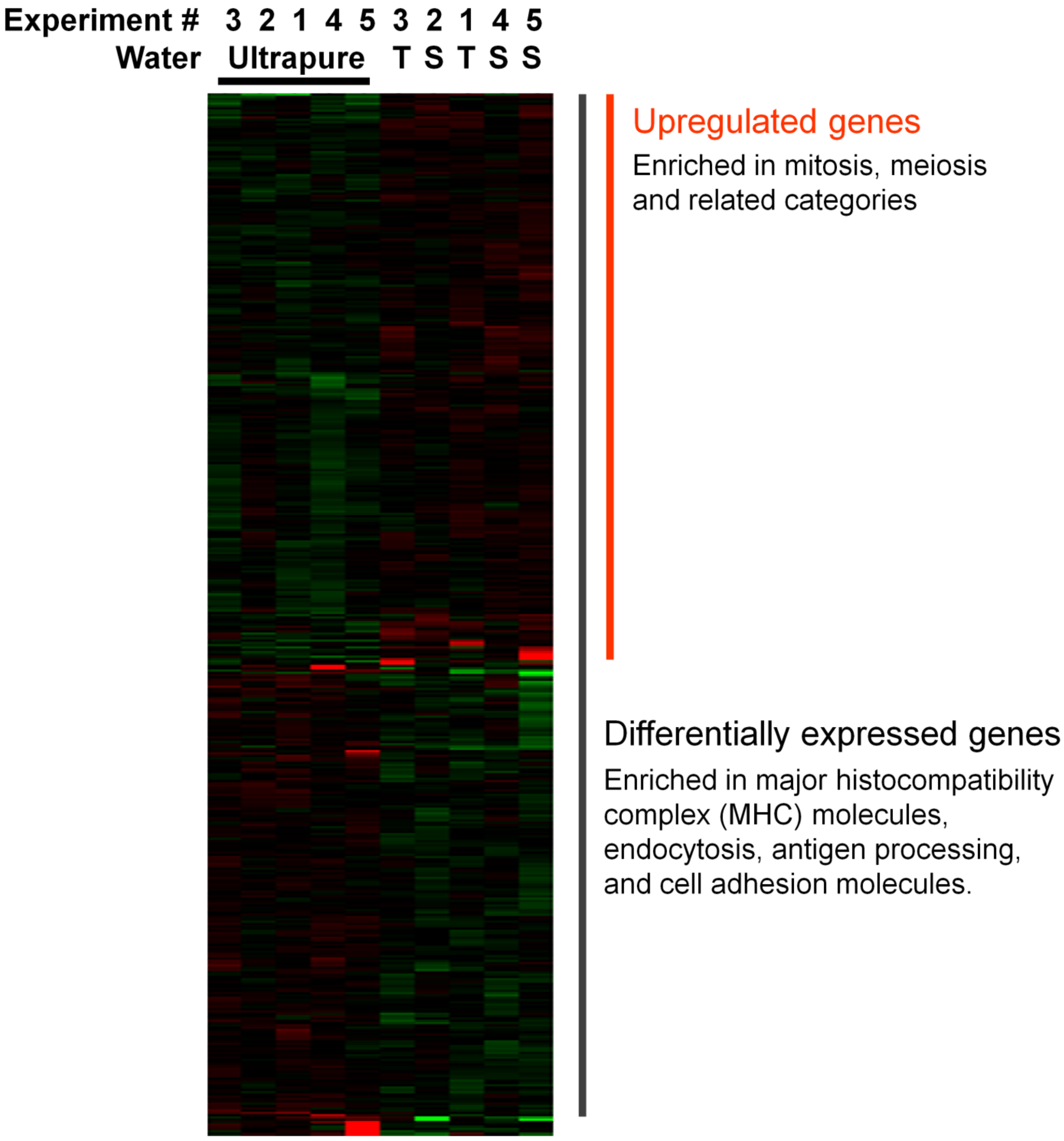

Among 15984 mRNAs with quantitative fluorescence signal (raw data ≥100), the data for genes with small variations among the ultrapure water groups are sorted into hierarchical clustering. “S” and “T” indicate high-silica water and tap water, respectively. The details of each experiment number were shown in Table 1.

Supplementary Table S2 − Differentially expressed genes with relevant enriched GO and pathways

Gene Symbol	antigen processing and presentation of peptide or polysaccharide antigen via MHC class II	MHC class II protein complex	antigen processing and presentation of exogenous peptide antigen via MHC class II	KEGG_ASTHMA	KEGG_TYPE_I_DIABETES_MELLITUS	KEGG_ALLOGRAFT_REJECTION	KEGG_AUTOIMMUNE_THYROID_DISEASE	KEGG_GRAFT_VERSUS_HOST_DISEASE	KEGG_ANTIGEN_PROCESSING_AND_PRESENTATION	KEGG_VIRAL_MYOCARDITIS	peptide antigen binding	KEGG_CELL_ADHESION_MOLECULES_CAMS	regulation of axonogenesis	platelet activation	gamma-aminobutyric acid signaling pathway	adenylate cyclase-activating G-protein coupled receptor signaling pathway	ST_GA13_PATHWAY	meiosis	PID_PLK1_PATHWAY	mitosis	chromosome, centromeric region	kinetochore	chromosome segregation	REACTOME_MITOTIC_PROMETAPHASE	MIPS_CENP_A_NAC_CAD_COMPLEX	decidualization	motile cilium	retinol metabolic process	axon part
H2-DMb1	+	+	+	+	+	+	+	+	+	+		+																	
H2-Ab1	+	+	+	+	+	+	+	+	+	+	+	+																	
H2-Eb1	+	+	+	+	+	+	+	+	+	+	+	+																	
H2-Q2					+	+	+	+	+	+		+																	
Tap1									+		+																		
Dmd										+																			
Sgcb										+																			
Cacna1a													+		+														
Cdh2												+	+																
Ssh1													+																
Cntnap2												+																	
Selplg												+																	
Cdh4												+																	
Itgb3														+															
Wnt3a													+	+															
Fhad1														+															
Htr4															+	+													
Gabra3															+														
Gna13														+		+	+												
Rock2																	+		+										
Cfb																	+												
Tbxa2r																	+												
Adora2a																+													
Glp1r																+													
Mnd1																		+											
Meig1																		+											
Ccna1																		+		+									
Cdk1																			+	+									
Cdk2																		+		+									
Nek2																		+		+	+	+	+						
Rad51																		+											
Aurka																			+	+									
Cenph																				+	+	+	+	+	+				
Lats2																				+									
Spag5																				+	+	+	+						
Cenpw																				+	+	+	+						
Cenpp																					+			+	+				
Spc24																			+	+	+	+		+					
Itgb3bp																				+	+	+		+	+				
Apitd1																				+	+	+		+	+				
Cdc20																			+	+				+					
Cenpe																			+	+	+	+							
Haus3																				+									
Mau2																				+			+						
Setdb2																				+			+						
Ghrl																										+			
Cited2																										+			
Ptgs2																										+			
Fsip1																											+		
Rsph9																											+		
Ropn1l																											+		
Catsper4																											+		
Lrat																												+	
Plb1																												+	
Rbp1																												+	
Dst																													+
Syn1																													+
Disc1																													+

Genes colored orange and blue were upregulated and downregulated by particles generated by the humidifier with tap water or high-silica water.
